# A Challenging Case of Refractory Hepatic Encephalopathy in a Postliver Retransplant Patient with Thrombosed Portal Vein: A Shunt for a Shunt

**DOI:** 10.1155/2023/6765788

**Published:** 2023-02-25

**Authors:** Fady Salama, Anna Christina Leyson, Malay Shah, Roberto Galuppo Monticelli

**Affiliations:** ^1^Department of Internal Medicine-Division of Digestive Diseases and Nutrition, University of Kentucky, Lexington, KY 40536, USA; ^2^Department of Surgery-Division of Transplant Surgery, University of Kentucky, Lexington, KY 40536, USA; ^3^Department of Radiology-Division of Vascular and Interventional Radiology, University of Kentucky, Lexington, KY 40536, USA

## Abstract

Hepatic encephalopathy (HE) is a frequent and serious complication of chronic liver disease. The mechanism of hepatic encephalopathy is not entirely clear. Hepatic encephalopathy is defined as brain dysfunction caused by liver insufficiency and/or portal-systemic blood shunting. It manifests as a wide spectrum of neurological or psychiatric abnormalities, ranging from subclinical alterations, detectable only by neuropsychological or neurophysiological assessment, to coma. Liver transplant (LT) is the definitive treatment for refractory hepatic encephalopathy. In this case, we present a challenging case of refractory hepatic encephalopathy in a postliver transplant patient with portal vein thrombosis and a splenorenal shunt treated with a novel technique to address his complex anatomy.

## 1. Case Presentation

**Figure 1:**
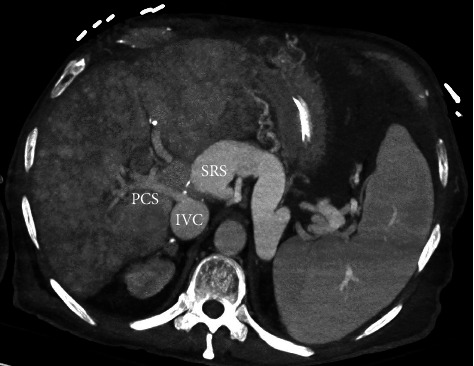
Maximum intensity projection contrast enhanced computed tomography showing the anatomical relationship between the transplant portocaval shunt (PCS), inferior vena cava (IVC), and the massive splenorenal shunt (SRS).

**Figure 2:**
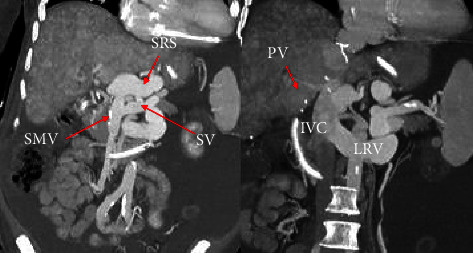
Contrast-enhanced CT showing both the large splenorenal shunt (SRS) draining the superior mesenteric vein (SMV) and splenic vein (SV) into the left renal vein (LRV) and the small transplant portal vein (PV) anastomosed to the inferior vena cava (IVC).

A 64-year-old Caucasian male who underwent an orthotopic liver transplant in 2008 for alcohol-related cirrhosis and was retransplanted in 2015 for chronic rejection. He had known chronic portal vein thrombosis and a large splenorenal shunt before his retransplant, with a patent confluence of the splenic and superior mesenteric veins. The decision was made to not create a venous conduit from the SMV to the donor portal vein due to concern of stealing from the large splenorenal shunt. Therefore, the portal vein reconstruction was performed by hemicaval transposition (portocaval shunt) with near ligation of the IVC superior to the portocaval shunt. Ligation of the IVC superior to the shunt allowed for caval blood flow to be directed antegradely through the donor portal vein. Five years after retransplant, he started presenting with covert hepatic encephalopathy [1], which progressed to recurrent severe HE with multiple admissions requiring an intubation despite optimizing medical treatment. All other causes of encephalopathy were ruled out. He did not have cirrhosis on imaging or MR elastography and had preserved graft synthetic function with normal bilirubin and INR. He had ascites attributed to portal hypertension from portal vein thrombosis. It was assessed that his recurrent HE is related to his large splenorenal shunt. Abdominal computed tomography (CT) showed no identifiable main portal vein, and there was cavo-portal transposition and a persistently large splenorenal shunt (Figure 1). Multidisciplinary discussion including hepatology, interventional radiology, and transplant surgery discussed that his venous reconstruction at the time of the retransplant was not conventional. The donor portal vein was anastomosed to the recipient IVC because the recipient portal vein was already thrombosed and ligated. Thus, there was no physical connection between splanchnic circulation and liver (Figure 2). The initial thought was that embolization of the splenorenal shunt would not improve venous inflow to the liver because there is no physical connection via a portal vein. So even if the splenorenal shunt is partially or completely embolized, liver circulation through the portal system will not improve, refractory HE may not improve at all, and portal hypertension may worsen.

**Figure 3:**
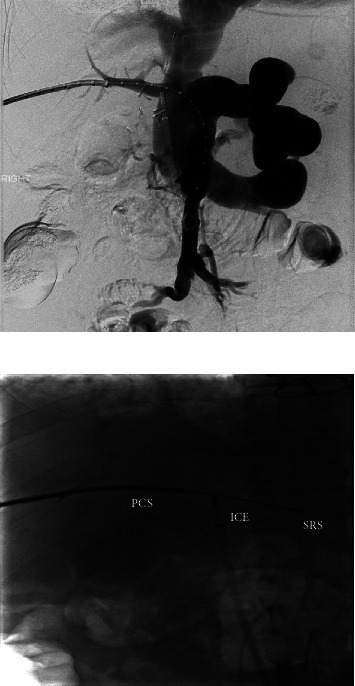
a & b: portal vein access. Challenging and relatively central access is due to diminutive portal vein branches. Intravascular ultrasound guidance.

**Figure 4:**
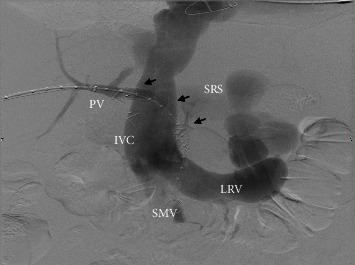
Newly established hepatopedal flow. Although, persistent high flow via the SRS.

After multiple multidisciplinary discussions, the patient underwent arterio- and venography which showed widely patent mesenteric arteries and a patent portocaval shunt, with retrograde flow within the portal vein into the IVC. There was no evidence of arterial portal shunting. Despite the IVC previously being nearly ligated during the retransplant procedure, the flow through the IVC at this time was robust. The superior mesenteric vein and splenic vein drain directly into the IVC via a large splenorenal shunt. Based on the angiogram and venogram, splenorenal shunt pressure was 12 mmHg; IVC pressure was 11 mmHg; right atrial pressure was 7-8 mmHg; and postsurgical portal vein pressure was 9 mmHg. It was felt that dilation and stenting of the portocaval shunt would not be effective because of the large flow of blood in the IVC. A few days later, the patient underwent porto-portacaval shunt via stent placement from the native portal vein stump to the transplant portocaval shunt (Figure 3). Predominant and retrograde flow remains extending from the portal system via the splenorenal shunt into the renal vein-IVC (Figure 4).

Three days later, the patient underwent embolization of the splenorenal shunt. The patient's mental status has subsequently improved significantly throughout the rest of his hospitalization and did not show any signs of overt hepatic encephalopathy. The patient was maintained on lactulose and rifaximin, eventually tapered off lactulose, and did not have further episodes of HE. One month later follow-up doppler showed that the new shunt was patent. Patient had no further hepatic encephalopathy related hospital admission or emergency room visit post intervention.

## 2. Discussion

Hepatic encephalopathy could be secondary to hepatic failure due to the liver synthetic dysfunction as in cirrhosis or acute liver failure; however, encephalopathy could be also secondary to spontaneous portosystemic shunts (SPSS) such as a large splenorenal shunt [2, 3]. The incidence of SPSS in patients with HE varies from 46% to 71%, and most patients with recurrent or persistent HE become refractory to medical therapy.

Liver transplantation (LT) is the definitive treatment for hepatic encephalopathy secondary to end-stage liver disease. A large portosystemic shunt may persist after a liver transplant which can cause hepatic encephalopathy with or without cirrhosis. Moreover, LT recipients can develop graft cirrhosis and portal hypertension related to SPSS which could be present before a live transplant or newly developed posttransplant. The impact of SPSS in the LT setting is broad and may be related to the portal flow steal phenomenon. It can be associated with postoperative complications due to a diminished portal flow which could lead to portal vein thrombosis [4]. It has been previously shown that in some situations, there is a need to ligate spontaneous portosystemic shunts at the time of transplantation to avoid portal flow steal [5, 6]. An attempt to recreate portal flow and splenorenal shunt embolization is an effective procedure when performed with expertise in interventional radiology in consultation with transplant surgery [7]. This is the first reported case of recreation of portal flow through stent placement from the native portal vein stump to the transplant portocaval shunt to re-establish portal vein flow before attempting splenorenal shunt embolization.

## 3. Conclusion

Refractory hepatic encephalopathy is uncommon but challenging in postliver transplant patients. LT recipients usually have graft cirrhosis before developing symptoms of HE. Patients with refractory hepatic encephalopathy postliver transplant require further workup, and spontaneous portosystemic shunts should be suspected. CT abdomen with contrast is recommended to assess portal circulation and screen for spontaneous portosystemic shunts like the splenorenal shunt. Further management should be based on multidisciplinary discussion including hepatology, transplant surgery, and interventional radiology. In patients with thrombosed portal veins, recreation of portal flow should be attempted, and embolization of the splenorenal shunt should be considered in such cases. More studies are needed to assess the benefits and risks of spontaneous portosystemic shunt ligation during transplant.

## Data Availability

The manuscript data used to support the findings of this study are available from the corresponding author upon request.

## References

[1] Kondo Y., Iwasa M., Kawaratani H. (2021). Proposal of Stroop test cut‐off values as screening for neuropsychological impairments in cirrhosis: a Japanese multicenter study. *Hepatology Research*.

[2] Choudhary N. S., Saigal S., Saraf N., Baijal S. S., Soin A. S. (2021). Recurrent hepatic encephalopathy due to surgically created shunt during living donor liver transplantation. *Journal of Clinical and Experimental Hepatology*.

[3] Tomás Pujante P., Jiménez Sánchez A. F., Iglesias Jorquera E., Pons Miñano J. A. (2018). Hepatic encephalopathy secondary to a splenorenal shunt that manifested a long time after a liver transplantation. *Revista Española de Enfermedades Digestivas*.

[4] Álvarez-López P., Campos-Varela I., Quiroga S. (2022). Spontaneous portosystemic shunt embolization in liver transplant recipients with recurrent hepatic encephalopathy. *Annals of Hepatology*.

[5] Philips C. A., Rajesh S., Augustine P., Padsalgi G., Ahamed R. (2019). Portosystemic shunts and refractory hepatic encephalopathy: patient selection and current options. *Hepatic Medicine: Evidence and Research*.

[6] Gomez Gavara C., Bhangui P., Salloum C. (2018). Ligation versus no ligation of spontaneous portosystemic shunts during liver transplantation: audit of a prospective series of 66 consecutive patients. *Liver Transplantation*.

[7] Ke Q., Wang Z., Huang X. (2022). Splenic vein embolization as a feasible treatment for patients with hepatic encephalopathy related to large spontaneous splenorenal shunts. *Annals of Hepatology*.

